# Correlation between CHA_2_DS_2_-VASc Score and Glaucoma Treatment and Prognosis

**DOI:** 10.1155/2018/8931430

**Published:** 2018-01-31

**Authors:** Yoav Y. Pikkel, Daniel Krebs, Vadim Igal, Adi Sharabi-Nov, Irena Epstein, Joseph Pikkel

**Affiliations:** ^1^Carmel Medical Center, Haifa, Israel; ^2^Faculty of Medicine, Bar-Ilan University, Safed, Israel; ^3^Tel Hai Academic College, Kiryat Shmona, Israel; ^4^Ophthalmology Department, Ziv Medical Center, Safed, Israel

## Abstract

**Purpose:**

To find if CHA_2_DS_2_-VASc scale can accurately predict the treatment, prognosis, and outcome for primary open-angle glaucoma (POAG).

**Patients and methods:**

A survey of 250,000 patient years was taken, using the records of the Ophthalmology Department at Ziv Medical Center. Data was collected regarding the retinal nerve fiber layer (RNFL), visual field (VF), line of treatment (LOT) of glaucoma, and all the data needed to accurately calculate CHA_2_DS_2_-VASc score for each patient.

**Results:**

Sixty-seven patients were included in the statistical analysis. The mean age was 72.5 years. The mean CHA_2_DS_2_-VASc score was 3.27 + −1.7. Positive Pearson's correlation coefficients were found for LOT and CHA_2_DS_2_-VASc score, 0.35, and for RNFL grade and CHA2DS2-VASc score, 0.37. The correlation was negative for RNFL width and CHA2DS2-VASc score, −0.35.

**Conclusions:**

CHA_2_DS_2_-VASc score was shown to be correlated with glaucoma. This correlation was manifested positively by the LOT needed to stop glaucoma progression, with higher CHA_2_DS_2_-VASc scores correlated with more aggressive treatment. Since glaucoma is a disease with a progressing nature, it is important to treat patients aggressively on one hand, while offering the most benign treatment as possible on the other hand. Modification of the CHA_2_DS_2_-VASc score could achieve an even higher correlation.

## 1. Introduction

Glaucoma is the second cause of blindness in the developed world [1]. Among the glaucoma subtypes, primary open-angle glaucoma (POAG) is the most common and is estimated to affect 53 million people worldwide by 2020 [2]. There are many risk factors for developing POAG, including age [3], race [4], hypertension [5], and diabetes [6]. Those risk factors are the same for the population in Israel. Uncontrolled POAG is primarily manifested by an elevated intraocular pressure (IOP) and irreversible loss of peripheral visual field (VF) [7]. Treatment goals in glaucoma are usually IOP < 25 mmHg and preservation of peripheral VF [8]. There are 4 main lines of treatment (LOT), as defined by the National Institute for Health (NIH) [9]. The first LOT is a monotherapy, for example, beta-blockers such as timolol, prostaglandins, carbonic-anhydrase inhibitors, alpha agonists, and adrenergic agonists. The second LOT comprises dual therapy that comprises 2 of the drugs in the first LOT. The third LOT is laser trabeculoplasty and the fourth LOT is a filtration surgery [9].

The CHA_2_DS_2_-VASc score was developed by cardiologists to assess the need of anticoagulant therapy after the detection of atrial fibrillation (AF). It is a modification of the previous CHADS_2_ score. The CHA_2_DS_2_-VASc score calculates the age of the patient and diseases such as chronic heart failure (CHF), hypertension, diabetes mellitus (DM), vascular diseases, and previous stroke or transient ischemic attack (TIA) [10]. The components of CHADS_2_ and CHA_2_DS_2_-VASc scoring scales are presented in Table 1. Although initially used to determine optimal anticoagulation therapy, the CHADS_2_ score and its modifications were found useful in identifying the prognosis of other diseases, including renal failure [11] and myocardial infarct [12], and recurrence of AF [13]. Since several components of the CHADS_2_ and CHA_2_DS_2_-VASc scores are independent risk factors for glaucoma, our hypothesis is that higher scores will be manifested by a higher LOT and more severe prognosis.

## 2. Patients and Methods

The Ophthalmology Department at Ziv Medical Center is the sole provider of ophthalmic care in the northeastern region of Israel, which includes the Galilee and Golan Heights, with approximately 250,000 people. The department offers all standard lines of treatments for glaucoma and a dedicated glaucoma clinic. Data from a computer output comprising all the patients who attended the glaucoma clinic at Ziv Medical Center during 12 consecutive months (1 January 2015–31 December 2015) were retrieved, in order to have full data as possible on disease and treatment. The study inclusion criterion was a documented case of POAG. In order to find the final LOT, patients without sufficient treatment for glaucoma were excluded. Thus, exclusion criteria were IOP > 25 mmHg, retinal detachment, and insufficient data in the patient chart.

Glaucoma was diagnosed as optic disk (OD) concaving of more than 0.5 or OD thinning of less than 5% of the average retinal nerve fiber layer (RNFL). POAG was diagnosed by a direct ophthalmoscope showing an open angle. Treatment was divided into 5 LOTs. Follow-up and watchful waiting were considered as LOT 0, monotherapy as LOT 1, dual therapy as LOT 2, laser trabeculoplasty as LOT 3, and filtration surgery as LOT 4. RNFL was measured with ocular coherence tomography (OCT), after a satisfying treatment, and classified into grades of deterioration. Intact RNFL, and no less than 5% of normal thickness in all parts of the OD, was considered as grade 0. RNFL thickness of 1–5% in at least 1/12 parts of the OD was considered grade 1, and <1% RNFL thickness in at least 1/12 parts of the OD was considered grade 2.

Cross-sectional data were extracted regarding glaucoma treatment, VF at initial diagnosis and after treatment, as measured by the Humphrey Visual Field Analyser, and RNFL thickness after treatment as measured with OCT by OTI, Canada. IOP was measured by Goldman's applanation tonometer. Data regarding best-corrected visual acuity (BCVA) was recorded. Parameters relevant to calculate CHADS_2_ and CHA_2_DS_2_-VASc scores, for example, age, chronic diseases such as CHF, hypertension, DM, vascular diseases, and previous CVA or TIA were also retrieved. Full CHADS_2_ and CHA_2_DS_2_-VASc scoring and algorithm are in Table 1.

### 2.1. Statistical Methods

Since there were more than 30 patients, normal distribution was assumed. For categorical variables, a summary is provided, which relates to sample size and relative frequencies. For continuous variables, summary tables are provided, including arithmetic means (M) and standard deviations (SD). The Kruskal-Wallis nonparametric tests were applied for examining the effects of clinical status on the quantitative demographic characteristics. Pearson correlations were applied for examining correlations between continuous variables, as were done in other studies where ordinal variables were aggregated into one score [14]. Statistical adjustment was done for intereye correlation. *P* value of 5% or less was considered statistically significant. The data were analyzed using the SPSS version 23 (SPSS Inc. Chicago, IL, USA).

This study was approved by Ziv Medical Center's institutional review board.

## 3. Results

Eighty-eight patients attended the glaucoma clinic during 12 consecutive months. The 13 patients who did not have glaucoma and the 6 who had a type of glaucoma other than POAG were excluded from the analysis. One patient had age-related macular degeneration and one had insufficient data in his chart regarding his glaucoma. Thus, 67 patients were included in the statistical analysis, with 130 eyes affected by glaucoma.

Patients attended the glaucoma clinic either for routine follow-up or due to the need for treatment or a specific exam. Routine follow-up patients (RFP) arrived due to severe glaucoma or to the lack of community clinics in their residential area. Community follow-up patients (CFP) usually attended the clinic due to cataract surgery, to undergo an OCT or as a consulting service to other hospital departments. In our cohort, 49 were RFP and 19 were CFP.

Thirty of the patients were men (45%) and 37 women. The mean age was 72.5 years (range: 45–92). The mean ± SD CHADS_2_ score was 1.95 + −1.2 (range: 0–5), and the mean ± SD CHA_2_DS_2_-VASc score was 3.27 + −1.7 (range: 0–7).

The treatment of 8 eyes was categorized as LOT 0, 44 as LOT 1 (40 with prostaglandin analogue and 4 with timolol malate), 66 as LOT 2, 8 as LOT 4, and 4 as LOT 4. The mean IOP with a stable and nonprogressive disease under continuous therapy or follow-up was 17.18 mmHg. Fifty-two eyes had 0 grade thinning, 24 had first grade thinning, and 41 had second grade thinning. The mean RNFL thickness was 86.83 + −15.46 (54–120).

The VF was assessed upon the first diagnosis of glaucoma and throughout follow-up. Comparing the VF at diagnosis and after treatment, a mean deterioration of 35 degrees (−10 to 145) was noted.

Mean CHADS_2_ and CHA_2_DS_2_-VASc scores, according to LOT, are presented in Table 2. Most vascular diseases were peripheral vascular diseases, that is, peripheral limb ischemia. None of the vascular disease patients were treated in LOT 0, 4 were treated with LOT 1, twenty were treated with LOT 2, and 5 were treated with LOT 3.

Pearson correlations of continuous variables with CHADS_2_ and CHA_2_DS_2_-VASc scores are presented in Table 3. Positive Pearson's correlation coefficients were found for LOT and CHA_2_DS_2_-VASc score *r* = 0.35 and for RNFL grade and CHA_2_DS_2_-VASc score *r* = 0.37. The correlation was negative for RNFL width and CHA_2_DS_2_-VASc score, *r* = −0.35. No significant correlation was found for RNFL width, RNFL grade, or LOT with CHADS_2_ scores; however, a tendency to an increasing correlation was observed from CHADS_2_ to CHA_2_DS_2_-VASc scores. No significant correlation was found for BCVA or VF with either CHADS_2_ or CHA_2_DS_2_-VASc scores.

The correlation of CHA_2_DS_2_-VASc scores with LOT was higher for the CFP than that with the RFP subgroup, *r* = 0.61 versus *r* = 0.25. Mean RNFL thicknesses were 0.53 and 1.08, respectively.

## 4. Discussion

Glaucoma is a multifactorial disease, with a pathogenesis closely related to several risk factors. For some patients, all risk factors are present and for others, some or none at all.

The current guidelines for glaucoma comprise several possible LOTs, with an empiric treatment given over several months until IOP < 25 mmHg and deterioration stops, or an ophthalmologist decides to try another treatment. This strategy ensures treatment with minimal drug dosage and combinations; yet it can be costly in time and in damage to the RNFL. Treatment challenges are further emphasized by the irreversible nature of the insult made by glaucoma to the RNFL and to the patients' VF. Thus, if a physician would have more direction in determining and tailoring treatment to patients, both time and RNFL could be saved and better patient outcomes achieved.

The CHADS_2_ score and its modifications were initially developed for use by cardiologists, but since their first implementation, their prognostic value for other conditions was realized [11–13]. Since these scores include several risk factors associated with the pathogenesis of glaucoma, it is reasonable to consider associations between the scores and factors relating to glaucoma findings. All CHADS_2_ scores and variations include hypertension, a major risk factor for glaucoma, and DM, another risk factor. We believe this to be at the base of the correlations observed herein.

We hypothesized that the greater the number of risk factors for glaucoma, the greater the potential for severe disease. Furthermore, the higher the scores on CHADS_2_ and CHA_2_DS_2_-VASc, the more chronically ill the patient, and the more likely to develop other diseases, which in turn might be more severe. We found CHA_2_DS_2_-VASc to correlate better than CHADS_2_ with glaucoma treatment and prognosis. This is evidently due to two main factors included in CHA_2_DS_2_-VASc and not in CHADS_2_. First, age over 75 years adds 2 points in CHA_2_DS_2_-VASc. Age is one of the most crucial risk factors for glaucoma, and the scoring allocated in CHA_2_DS_2_-VASc to this criterion is probably one of the reasons for the superiority of this scale in relationship to glaucoma. Second, the CHA_2_DS_2_-VASc score includes vascular disease. A single patient can receive one point for any vascular disease and 2 points for stroke or TIA, which may also have a vascular etiology. Thus, up to 3 points might be accumulated due to a vascular disease. This compares with the CHADS_2_ score, in which the same disease awards 2 points. Though not a major risk factor, vascular disease is associated with glaucoma [15].

Our findings of greater correlation of CHA_2_DS_2_-VASc scores with LOT and lower mean RNFL thickness, among community follow-up than routine follow-up patients suggest that patients who were adherent to the therapy prescribed and attended the community clinic regularly had a milder disease. Although the number of community follow-up patients was relatively small in this cohort, we suspect that the CHA_2_DS_2_-VASc score can help in selecting optimal LOT for these patients.

The VF was relatively intact among the cohort. No correlation was found between VF and either CHADS_2_ or CHA_2_DS_2_-VASc scores. We suspect that since glaucoma is a relatively slowly progressing disease, proper treatment and close follow-up can halt disease progression, with minimal damage to the VF.

### 4.1. Study Limitations

This cohort comprised 67 patients with 130 affected eyes. Considering that the population attending Ziv Medical Center is about 250,000, underdiagnosis is probable. There are several reasons for such. Due to the nature of this study, patients with glaucoma who are treated other than at the glaucoma clinic would not be included. The population in northeastern Israel is rural and generally of a low socioeconomic status. Such populations tend to less frequently attend a hospital [16, 17]. Furthermore, considering the slow disease progression of glaucoma and low hospital attendance, a patient may arrive at a clinic when vision is almost completely lost, and no treatment can be offered, thus causing underdiagnosis as well. In addition, patients might choose to attend a clinic located at a further distance, due to a misconception of the capabilities of the Ophthalmology Department at Ziv Medical Center, thus constituting yet another reason for underdiagnosis.

A selection bias may be relevant to our cohort. Usually, RFP are of a more severe disease than CFP. In our cohort, CFP presented with better correlation between CHA_2_DS_2_-VASc score and LOT. Thus, it is our notion that a cohort that would comprise solely CFP patients would show even better correlation.

## 5. Conclusion

CHA_2_DS_2_-VASc score was shown to be correlated with glaucoma. This correlation was manifested positively by the LOT needed to stop glaucoma progression, with higher CHA_2_DS_2_-VASc scores correlated with more aggressive treatment. The relationship is further manifested in the correlation between thinning of RNFL and higher CHA_2_DS_2_-VASc scores. Since glaucoma is a disease with a progressing nature, it is important to treat patients aggressively on one hand, while offering the most benign treatment as possible on the other hand.

It is our notion that the use of the CHA_2_DS_2_-VASc score can help skip one or even two LOTs in glaucoma treatment. The CHA_2_DS_2_-VASc score showed a strong correlation to LOT with CFP. From this, we expect that in a compliant patient, this score may be particularly useful for the ophthalmologist. The CHA_2_DS_2_-VASc score showed superiority to the CHADS_2_ score. We expect that modification of the CHA_2_DS_2_-VASc score could achieve an even higher correlation; this is a direction for future study.

## Figures and Tables

**Table 1:** The components of CHADS_2_ and CHA_2_DS_2_-VAS_c_ scores.

Criteria	CHADS_2_ score points	CHA_2_DS_2_-VASc score points
Congestive heart failure	1	1
Hypertension	1	1
Age > 65 years	1	1
Age > 75 years	0	1
Diabetes mellitus	1	1
Stroke/transient ischemic attack	2	2
Thromboembolic event without stroke	0	1
Vascular disease	0	1

**Table 2:** Means, standard deviations, and differences of the CHADS scores according to the LOT of the study patients.

LOT (treatment)	CHADS_2_		CHA_2_DS_2_-VASc	
	M	SD	M	SD
0 (follow-up only)	1.0^b^	0.8	1.5^b^	0.9
1 (monotherapy)	1.9^ab^	1.2	3.0^ab^	1.5
2 (combined therapy)	1.9^ab^	1.2	3.5^ab^	1.7
3 (laser)	2.8^a^	1.0	4.6^a^	1.4
4 (surgery)	3.3^a^	2.1	4.5^a^	1.9
*P*	0.028		0.002	

Note: ^a–b^Different letters in each column represent significant differences between the means (*p* < 0.05). CHADS_2_ = congestive heart failure, hypertension, age > 65, diabetes mellitus, stroke; CHA_2_DS_2_-VASc = CHADS_2_ + gender (female) + age ≥ 75 + thromboembolic event + vascular disease; LOT = line of treatment; SD = standard deviation.

**Table 3:** Pearson correlations of CHADS_2_ and CHA_2_DS_2_-VASc scores with different variables.

	CHADS_2_	CHA_2_DS_2_-VASc
VF	0.140	0.055
RNFL	−0.247^∗^	−0.356^∗∗^
VA	−0.206	−0.345^∗∗^
Grade	0.262^∗∗^	0.380^∗∗∗^
Age	0.435^∗∗^	0.610^∗∗∗^
LOT		0.350^∗^

^∗^ *p* < 0.05, ^∗∗^*p* < 0.01, ^∗∗∗^*p* < 0.001. VF = visual field; RNFL = retinal nerve fiber layer average width; VA = visual acuity; Grade = RNFL grade; CHADS_2_ = congestive heart failure, hypertension, age > 65, diabetes mellitus, stroke; CHA_2_DS_2_-VASc = CHADS_2_ + gender (female) + age ≥ 75 + thromboembolic event + vascular disease.
